# Effects of Rosemary, Citronella, and Tea Tree Essential Oils on Fermentation Characteristics and Aerobic Stability of Sorghum Silage

**DOI:** 10.1002/fsn3.72258

**Published:** 2026-09-25

**Authors:** José Ribeiro Meirelles Júnior, Tadeu Silva de Oliveira, Ismael Nacarati da Silva, Elvanio José Lopes Mozelli Filho, Elon Souza Aniceto, Malony Tavares Lima, Jéssica Luizi de Souza Andrade, Breno Augusto Vaz Santos

**Affiliations:** ^1^ Laboratory of Animal Science State University of Northern of Rio de Janeiro Campos dos Goytacazes Brazil; ^2^ Capixaba Institute for Research Technical Assistance and Rural Extension Vitória Brazil; ^3^ Laboratory of Biotechnology State University of Northern of Rio de Janeiro Campos dos Goytacazes Brazil

**Keywords:** additive, antimicrobial, preservation, *Sorghum bicolor* (L.)

## Abstract

This study evaluated the effects of increasing doses of rosemary, citronella, and tea tree essential oils on fermentation characteristics, nutritional value, microbial count, losses, and aerobic stability of sorghum silage. A completely randomized design was used in a 3 × 4 factorial scheme, consisting of three essential oils (citronella, rosemary, and tea tree) and four inclusion levels: 0 (control silage without additive), 500, 1000, and 2000 mg kg^−1^ of fresh forage, with four replicates per treatment. The inclusion of rosemary essential oil increased crude protein content (*p* = 0.006), while reducing ammonia nitrogen (*p* = 0.029) and butyric acid concentration (*p* < 0.001). Increasing essential oil levels resulted in a quadratic response for silage pH (*p* = 0.003) and a linear decrease in lactic acid concentration (*p* = 0.018). Additionally, higher inclusion levels led to reduced dry matter recovery (*p* = 0.001) and increased lactic acid bacteria counts (*p* = 0.016). Regarding aerobic stability, the addition of 1000 mg kg^−1^ of citronella essential oil increased stability by 96 h. Essential oil treatments also influenced silage pH dynamics during aerobic exposure (*p* < 0.05). Overall, essential oils modified the fermentation and aerobic stability of sorghum silage. Rosemary essential oil was associated with lower NH_3_‐N and butyric acid concentrations, whereas higher inclusion levels increased LAB counts.

## Graphical abstract

Rosemary, citronella, and tea tree essential oils were evaluated as natural additives for sorghum silage at four inclusion levels (0, 500, 1000, and 2000 mg kg^−1^ of fresh forage). Rosemary essential oil improved fermentation by increasing crude protein while reducing ammonia nitrogen and butyric acid concentrations. Citronella essential oil at 1000 mg kg^−1^ enhanced aerobic stability by 96 h, demonstrating the potential of essential oils as natural alternatives to improve silage quality and preservation.

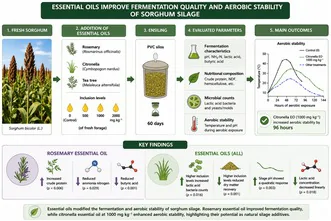

## 1 Introduction

Silages containing greater amounts of residual water‐soluble carbohydrates, such as sorghum silage, may be more susceptible to aerobic deterioration after oxygen exposure (Weinberg et al. 1993), as yeasts and other aerobic microorganisms can metabolize residual sugars and fermentation acids, leading to increases in temperature and pH, as well as nutrient losses (Shan et al. 2021). In this context, the adoption of additives has been widely explored as a strategy to improve fermentation dynamics, reduce nutrient losses, and enhance overall silage quality. Among the available additives, antifungal compounds are especially important during the feed‐out phase, when silage is exposed to oxygen, as they help limit the growth of yeasts and molds and prevent undesirable fermentative processes.

Essential oils (EO) have emerged as promising natural additives due to their antimicrobial properties, and previous studies have reported their potential to inhibit fungal growth in whole‐plant corn silage (Kung Jr et al. 2008; Chen et al. 2023; Silva et al. 2025; Andrade et al. 2026). Previous studies have reported their activity against a wide range of microorganisms associated with silages produced from different forages, including alfalfa, barley, corn, Italian ryegrass, sugarcane, and Sorghum (Chaves et al. 2012; Foskolos et al. 2016; Turan and Soycan‐Önenç 2018; Cantoia Jr et al. 2020; Aniceto et al. 2024; Silva et al. 2024). However, their effectiveness is not constant and may vary according to their chemical composition and the level of inclusion (Liang et al. 2023). In addition, environmental conditions such as temperature and water availability can influence the synthesis of secondary metabolites in plants, thereby altering the composition of essential oils (Eloutassi et al. 2023).

As a result, essential oils have attracted attention for their antimicrobial potential in silage preservation. Rosemary (*Salvia rosmarinus* Spenn.) produces an essential oil rich in 1,8‐cineole (40.55%–45.10%), camphene (17.40%–19.35%), and α‐pinene (10.73%–15.06%), compounds associated with antibacterial and antifungal activity (Atti‐Santos et al. 2004). Citronella ( *Cymbopogon winterianus* ) contains high concentrations of citronellal (40%), geraniol (27.44%), and citronellol (10.45%), which are responsible for its antimicrobial effects (Nascimento et al. 2011). Likewise, tea tree (*Melaleuca alternifolia*) contains terpinen‐4‐ol (42%), γ‐terpinene (19%), and α‐terpinene (10%), compounds linked to its antimicrobial properties (Yu et al. 2015). However, the composition of essential oil varies considerably depending on the climate, soil, harvest period, extraction method, cultivar, and other factors (Barra 2009).

Based on the antimicrobial properties of essential oils, we hypothesized that rosemary, citronella, and tea tree essential oils could modulate the fermentation process and aerobic stability of sorghum silage, with responses depending on the type and inclusion level of the essential oil. Therefore, this study evaluated the effects of increasing concentrations of these essential oils on the fermentative profile, losses, nutritional value, microbial counts, and aerobic stability of sorghum silage.

## 2 Materials and Methods

### 2.1 Location and Silo Type

The experiment was conducted in Campos dos Goytacazes, Rio de Janeiro, Brazil (21°45′45″ S, 41°17′06″ W; 8 m above sea level). According to the Köppen–Geiger classification, the region presents an Aw climate (tropical savanna climate with a dry winter) (Alvares et al. 2013). Throughout the experimental period, ambient temperature remained within the expected seasonal range, with no evidence of thermal anomalies.

All procedures involving experimental protocols were approved by the Animal Care and Use Committee of the Universidade Estadual do Norte Fluminense—Darcy Ribeiro (protocol no. 503/2021).

The forage material consisted of sorghum ( *Sorghum bicolor* (L.) Moench), hybrid BRS 658, characterized by drought tolerance, resistance to lodging, satisfactory foliar sanitary status, and tolerance to downy mildew (*Peronosclerospora sorghi*). The experimental area was established under conventional tillage, with rows spaced 0.70 m apart and a seeding depth of approximately 5 cm. Fertilization management was performed according to soil test recommendations, with the application of 250 kg ha^−1^ of fertilizer, following the guidelines of the Liming and Fertilization Manual for the State of Rio de Janeiro. Liming was not required. Sowing was carried out manually, at a seeding rate of six viable seeds per meter of row.

Harvesting was performed manually at a mean dry matter concentration of 380.36 g kg^−1^ (fresh basis). Subsequently, the forage was mechanically processed using a stationary forage chopper (JF Maxxium, JF Agricultural Machinery LTDA, Brazil), targeting a theoretical length of cut of approximately 1.5 cm.

Ensiling was conducted using laboratory‐scale silos constructed from polyvinyl chloride (15 cm diameter × 50 cm high), with an internal volume of approximately 8.84 L. Each silo was fitted with a Bunsen‐type valve to allow gases generated during fermentation to escape and contained approximately 600 g of oven‐dried sand at the base, separated from the ensiled mass by a cotton fabric layer to enable effluent recovery. The forage was compacted to achieve a packing density of 600 kg m^−3^ (fresh‐matter basis), resulting in approximately 5.3 kg of fresh forage per silo. The silos were stored under controlled ambient conditions (25°C ± 3.2°C) for 60 days during fermentation.

### 2.2 Experimental Design, Treatments, and Essential Oil Characterization

A completely randomized design was adopted in a 3 × 4 factorial arrangement, with three essential oil sources (citronella, rosemary, and tea tree) and four inclusion levels (0, 500, 1000, and 2000 mg kg^−1^ of fresh forage). Independent untreated control silages (0 mg kg^−1^) were prepared for each essential oil source, whereas the remaining treatments involved applying essential oils at increasing concentrations relative to the ensiled mass (on a fresh‐matter basis). Each treatment combination was evaluated with four biological replicates.

The essential oils were uniformly applied by spraying 50 mL of ethanol onto the forage mass prior to silo sealing, ensuring adequate distribution within the ensiled material. The inclusion rates were defined based on the antimicrobial responses reported by Chaves et al. (2012), with the highest inclusion level corresponding to the maximum dose previously evaluated by Foskolos et al. (2016).

All essential oils were commercially sourced and obtained through steam distillation (Ferquima Indústria e Comércio LTDA, Vargem Paulista, São Paulo, Brazil; CAS Number: 8000‐25‐7 [Rosemary essential oil]; CAS Number: 68647‐73‐4 [Tea tree]; and CAS Number: 8000‐29‐1 [citronella]). The physicochemical characteristics and major constituents of each oil were as follows. Rosemary essential oil presented a density of 0.90–0.93 g mL^−1^ and a refractive index of 1.460–1.475 (at 20°C), with 1,8‐cineole (40%), camphor (15%), α‐pinene (13%), and β‐pinene (7%) as the predominant compounds. Tea tree essential oil exhibited density values from 0.885 to 0.906 g mL^−1^ and refractive index between 1.470 and 1.482 (at 20°C), being mainly composed of terpinen‐4‐ol (41%), γ‐terpinene (21%), and α‐terpinene (9%). Citronella essential oil showed density ranging from 0.875 to 0.895 g mL^−1^ and refractive index between 1.463 and 1.473 (at 20°C), with β‐citronellal (32.7%), geraniol (28.9%), and citronellol (9.6%) as its principal constituents.

### 2.3 Chemical Composition and In Vitro Degradability

Samples of fresh forage and silage (approximately 500 g) were collected and dried in a forced‐air circulation oven at 55°C for 72 h. Subsequently, the dried material was processed in a Wiley‐type mill (Tecnal, Piracicaba, SP, Brazil) using a 1‐mm screen to obtain a homogeneous sample for laboratory analyses.

Chemical composition was determined according to standardized procedures. Dry matter (DM; method 967.03), crude fat (CF; method 2003.06 using hexane as the extraction solvent), ash (method 942.05), and crude protein (CP; calculated as N × 6.25) were quantified following the protocols described by Association of Official Analytical Chemists (AOAC 2019). The neutral detergent fiber content, expressed exclusive of residual ash (aNDFom), was determined using sodium sulfite and thermostable α‐amylase, according to INCT‐CA method F‐001/1 (Detmann et al. 2012). Acid detergent fiber (ADF) and lignin (sa) were determined following INCT‐CA methods F‐003/1 and F‐005/1, respectively (Detmann et al. 2012). Non‐fiber carbohydrates (NFC) were estimated by difference, considering the proportions of CP, CF, ash, and NDF in the sample (NFC g/kg = 1000 − [CP + CF + Ash + NDF]). Hemicellulose and cellulose fractions were obtained by sequential subtraction, calculated as the difference between NDF and ADF, and between ADF and lignin, respectively, with all components expressed on a dry matter basis (g kg^−1^ DM).

In vitro degradability of dry matter and neutral detergent fiber was determined according to the two‐stage technique described by Goering and Van Soest (1970). Analyses were performed in triplicate. Rumen fluid was obtained from three cannulated sheep fed a total mixed ration composed of sorghum silage and concentrate (ground corn and soybean meal), formulated to contain 180 g kg^−1^ CP and 520 g kg^−1^ NDF (DM basis), in addition to a mineral supplement. The ruminal fluid was mixed in a blender for 30 s to homogenize the liquid and solid phases. The homogenized material was filtered through four layers of gauze in 2 L Erlenmeyer flasks connected to a CO_2_ hose and kept in a water bath at 39°C. The buffer solution was prepared according to McDougall (1948). Samples (200 mg, standard deviation = 10 mg) were added to amber flasks (100 mL) with 50 mL of the previously prepared inoculum (1:4 ratio, ruminal fluid and buffer solution) and were hermetically sealed with rubber stoppers. After 48 h of incubation under controlled conditions, the flasks were removed and the contents were washed with hot distilled water (> 90°C) to interrupt microbial activity.

The undigested residue was recovered using pre‐weighed filter paper (Whatman, 150 mm diameter), dried at 55°C for 24 h, then oven‐dried at 105°C for 16 h, and subsequently weighed to determine residual dry matter. The remaining material was further analyzed for NDF concentration to estimate the undegraded fiber fraction. Degradability for DM and NDF was calculated based on the relationship between incubated substrate, residual fraction, and blank correction, expressed on a mass basis.

### 2.4 Fermentative Losses

Fermentative losses were quantified using mass‐balance procedures, accounting for gas and effluent outputs during ensiling, as described by Jobim et al. (2007).

Gas losses (GL) were estimated from the difference between silo weight at sealing and at opening, relative to the amount of ensiled dry matter, as described in Equation (1):

(1) $$\mathrm{GL}=\left(\text{WSWE}-\text{WSWO}\right)/\mathrm{EDM}$$

where GL represents gas losses (g kg^−1^ DM); WSWE corresponds to the weight of the silo at ensiling (g); WSWO is the silo weight at opening (g); and EDM is the amount of ensiled dry matter (kg).

Effluent losses (EL) were determined based on the variation in empty silo weight before and after the ensiling period, as presented in Equation (2):

(2) $$\mathrm{EL}=\left(\text{ESWO}-\text{ESWE}\right)/\mathrm{EDM}$$

where EL denotes effluent losses (g kg^−1^ DM); ESWO is the empty silo weight at opening (g); ESWE is the empty silo weight at sealing (g); and EDM corresponds to the ensiled dry matter (kg).

Dry matter recovery (DMR) was calculated as the proportion of dry matter remaining at silo opening in relation to the initial ensiled dry matter, according to Equation (3):

(3) DMR=ODM/EDM

where DMR is expressed as g kg^−1^ DM; ODM represents the dry matter recovered at opening (g); and EDM is the initial ensiled dry matter (g).

The Flieg score was used as an indicator of silage quality, based on the relationship between dry matter content and pH, following the equation proposed by Kilic (1986):

(4) $${\text{Flieg}}^{'}s\;\text{Score}=200+\left(2\times \%\mathrm{DM}-15\right)-40\times \mathrm{pH}$$

### 2.5 Fermentation Parameters and Microbial Count

Fermentation end products were quantified using aqueous extracts obtained from fresh silage samples, prepared according to the procedure described by Kung Jr et al. (1984) water extract method. The pH of the extracts was immediately measured using a digital potentiometer (Tecnal, Piracicaba, SP, Brazil). An aliquot of 15 mL of the extract was filtered and acidified with 100 μL of 0.036 N sulfuric acid to stabilize fermentation metabolites. The samples were subsequently stored under freezing conditions until further analysis. Concentrations of lactic, acetic, propionic, and butyric acids, as well as ethanol, were determined by high‐performance liquid chromatography (HPLC) using an LCMS‐2020 system (Shimadzu, Tokyo, Japan). Separation was achieved using a Rezex RCM‐Monosaccharide Ca^2+^ (8%) column (300 × 7.8 mm), with ultrapure water as the mobile phase at a constant flow rate of 0.7 mL min^−1^. The column temperature was maintained at 60°C, and compounds were detected via refractive index. Ammonia nitrogen (NH_3_‐N) concentration was determined by alkaline distillation using magnesium oxide, following the method proposed by Fenner (1965) ammonia nitrogen method.

For microbial count, 25 g of fresh silage were aseptically collected and homogenized with 225 mL of sterile saline solution (0.85% NaCl) for 60 s. From this initial suspension, successive decimal dilutions were prepared, ranging from 10^−1^ to 10^−6^, with 1000 μL plated for each dilution. Aliquots from appropriate dilutions were plated onto selective culture media to quantify distinct microbial groups. Lactic acid bacteria (LAB) were enumerated on Man, Rogosa, and Sharpe (MRS) agar, incubated at 37°C for 48 h. Enterobacteria were cultivated on violet red bile agar under the same temperature for 24 h. Fungal populations (yeasts and molds) were determined using potato dextrose agar, incubated at 25°C for 4 days (Siqueira 1995). Microbial populations were expressed as colony‐forming units per gram of fresh matter (cfu g^−1^). Prior to statistical analysis, data were log_10_‐transformed to ensure normalization and homogeneity of variance.

### 2.6 Aerobic Stability

After silo opening, approximately 2.0 kg of silage from each experimental unit was placed into 5.0 kg plastic containers for evaluation of aerobic stability. The material was exposed to air for 7 days under ambient conditions. Environmental temperature was continuously monitored at 6‐h intervals using data loggers (Log 110 EXF, Inconterm, Brazil), with an average of 25°C ± 2.3°C throughout the evaluation period. In parallel, silage pH was measured daily to monitor changes during aerobic exposure. Aerobic stability was determined as the time (hours) from air exposure until the silage temperature exceeded ambient temperature by 2°C, according to the methodology described by Ranjit and Kung Jr (2001).

### 2.7 Statistical Analysis

Prior to statistical analysis, data were evaluated for compliance with the assumptions of normality and homogeneity of variances. Normality of residuals was assessed using the Shapiro–Wilk test implemented in the SAS UNIVARIATE procedure, whereas homogeneity of variances was evaluated using Levene's test through the SAS GLM procedure [HOVTEST = LEVENE option] (SAS University Edition; SAS Institute Inc., Cary, NC, USA). Subsequently, the data were analyzed using polynomial regression and Tukey's multiple‐comparison test with the MIXED procedure in SAS. The essential oil dose was treated as a quantitative factor and evaluated using polynomial regression to assess linear and quadratic responses. Essential oil source was treated as a qualitative factor, and treatment means were compared using Tukey's multiple‐comparison test at a significance level of *α* = 0.05. A tendency was considered when 0.10 > *p* > 0.05.

The following statistical model was used:

$$Y_{\mathrm{ijk}}=\mu +\alpha_{i}+\beta_{j}+{\alpha \beta }_{\mathrm{ij}}+e_{\mathrm{ijk}}$$

In which: $Y_{\mathrm{ijk}}$ is the value observed for the variable under study referring to the *k*‐th repetition of the combination of the *i*‐th factor level *α* with the *j*‐th factor level *β*; μ is the mean of all experimental units for the variable under study; $\alpha_{i}$ are the essential oils effects with *i* = 1,2, 3; $\beta_{j}$ are the levels of essential oils in the silages with *j* = 1, 2, 3, 4; ${\alpha \beta }_{\mathrm{ij}}$ is the interaction between the essential oils and the levels; $e_{\mathrm{ijk}}$ is the error associated with the observation $Y_{\mathrm{ijk}}$. The fixed effects are the μ, $\alpha_{i}$, $\beta_{j}$, and ${\alpha \beta }_{\mathrm{ij}}$. The random effect is $e_{\mathrm{ijk}}$.

The effects of increasing inclusion levels of essential oils were further evaluated by regression analysis within the same mixed‐model framework. For variables associated with aerobic stability and pH dynamics, time‐course measurements were analyzed as repeated measures, using the appropriate covariance structure, and evaluated via regression at the 5% significance level. Variables measured repeatedly over time were analyzed using the PROC MIXED procedure of SAS. The experimental unit consisted of the field plot associated with each individual silo (random effect). Evaluation time was treated as a fixed effect because the sampling points were predefined in the experimental protocol. Repeated measurements within the same experimental unit were modeled using a compound symmetry covariance structure, selected based on the corrected Akaike information criterion (AICc). Degrees of freedom were estimated using the Kenward–Roger method. The following statistical model was used:

$$Y_{\text{ijkl}}=\mu +\alpha_{i}+\beta_{j}+\tau_{k}+{\alpha \beta }_{\mathrm{ij}}+{\alpha \tau }_{\mathrm{ik}}+{\beta \tau }_{\mathrm{jk}}+{\alpha \beta \tau }_{\mathrm{ijk}}+S_{l\left(\mathrm{ij}\right)}+e_{\text{ijkl}}$$

In which: $Y_{\text{ijkl}}$ is the value observed for the variable under study referring to the *l*‐th replicate of the combination of *i*‐th factor level *α* with the *j*‐th factor level *β* in the *k*‐th hour; μ is the mean of all experimental units for the variable under study; $\alpha_{i}$ are the essential oils effects *i* = 1,2, 3; $\beta_{j}$ are the levels of essential oils in the silages with *j* = 1, 2, 3, 4; $\tau_{k}$ is the fixed effect of the evaluation hours with *K* = 0, 24, …, 144 for pH and 0, 8, 16, …, 168 for temperature; ${\alpha \beta }_{\mathrm{ij}}$ is the interaction between the essential oils and the levels; ${\alpha \tau }_{\mathrm{ik}}$ is the interaction between essential oils and evaluation hours; ${\beta \tau }_{\mathrm{jk}}$ is the interaction between levels of essential oils and evaluation hours; ${\alpha \beta \tau }_{\mathrm{ijk}}$ is the interaction between essential oils, levels of essential oils, and evaluation hours; $S_{l\left(\mathrm{ij}\right)}$ is random effect of silo nested within each treatment combination; $e_{\text{ijkl}}$ is the error associated with observation $Y_{\text{ijkl}}$.

## 3 Results

No significant interaction between essential oil type and inclusion level was observed for any of the evaluated variables (*p* > 0.05); therefore, the main effects are presented separately.

With respect to chemical composition, silages treated with rosemary essential oil exhibited higher CP content (*p* = 0.006) compared to the other essential oil sources, whereas no differences were detected for the remaining variables (*p* > 0.05) (Table 1). When considering the effect of inclusion levels, organic matter (OM; *p* = 0.049) and CP (*p* < 0.001) were influenced by increasing concentrations of essential oils (Table 1).

**TABLE 1:** Effects of essential oils and different levels on the chemical composition and in vitro degradability of sorghum silage.

Variables	Essential oils				SEM	*p*
	Sorghum	Tea tree	Citronella	Rosemary		
DM	380.36	365.11	372.9	367.95	0.409	0.897
OM	320.78	296.97	303.53	300.26	0.316	0.918
CP	47.79	46.78^b^	49.15^b^	51.87^a^	0.152	0.006*
NDF	685.26	696.78	703.91	679.23	1.355	0.218
Lig	15.46	19.51	14.11	16.45	0.271	0.153
NFC	186.74	154.82	143.66	164.63	1.031	0.412
Hem	226.94	239.37	240.69	230.95	0.582	0.415
Cell	442.86	437.89	445.76	431.82	0.700	0.503
IVDMD	487.67	520.75	516.69	530.6	0.762	0.543
IVNDFD	570.48	520.17	509.09	517.42	0.623	0.462

Variables	Levels				SEM	*p*
	0	500	1000	2000		
DM	345.11	363.46	385.32	380.73	2.074	0.173
OM	269.98	297.92	318.14	314.97	2.353	0.049^†^
CP	42.28	53.82	50.13	50.83	0.504	< 0.0001^†^
NDF	707.5	691.03	683.84	690.85	1.024	0.522
Lig	14.51	16.61	15.75	19.91	0.232	0.370
NFC	139.22	151.61	168.58	158.07	1.293	0.416
Hem	241.11	237.43	233.59	235.91	0.327	0.863
Cell	451.88	432.53	434.51	435.04	0.966	0.466
IVDMD	513.11	547.76	515.34	514.5	1.810	0.071
IVNDFD	505.04	529.87	515.38	511.95	1.033	0.139

*Note:* Sorghum = sorghum plant before ensiling; 0 = control (0 mg); 500 = 500 mg/kg of mass forage; 1000 = 1000 mg/kg of mass forage; 2000 = 2000 mg/kg of mass forage.

Abbreviations: Cell, cellulose; CP, crude protein; DM, dry matter; Hem, hemicellulose; IVDMD, in vitro dry matter degradability; IVNDFD, in vitro neutral detergent fiber degradability, all expressed as g/kg, except DM expressed as as‐fed; Lig, lignin; NDF, neutral detergent fiber; NFC, non‐fibrous carbohydrate (1000 − CP − EE − Ash − NDF); OM, organic matter; SEM, standard error of the mean.

* Means followed by different letters in a line differ significantly by the Tukey test (*p* < 0.05).

^†^ Regression analysis. OM = 222.57 ± 33.52 + 54.40 ± 30.58 × Levels − 7.77 ± 6.02 × Levels^2^; CP = 30.22 ± 4.18 + 15.76 ± 3.82 × Levels − 2.71 ± 0.75 × Levels^2^.

The fermentative profile was also influenced by essential oil treatments (*p* < 0.05). Specifically, rosemary essential oil resulted in lower NH_3_‐N (*p* = 0.029) and butyric acid concentrations (*p* < 0.001) (Table 2). In addition, increasing inclusion levels promoted a quadratic response in silage pH (*p* = 0.003), while lactic acid concentration decreased linearly (*p* = 0.018) (Table 2).

**TABLE 2:** Effects of essential oils and different levels on fermentative profile of sorghum silage.

Variables	Essential oils			SEM	*p*
	Tea tree	Citronella	Rosemary		
T, °C after opening the silo	23.94	24.09	23.94	0.009	0.958
pH after opening the silo	4.57	4.51	4.47	0.005	0.073
NH_3_‐N, g/kg CP	0.71^b^	0.76^a^	0.66^c^	0.005	0.029*
Lactic acid, g/kg DM	76.50	75.64	77.53	0.094	0.916
Acetic acid, g/kg DM	16.10	16.21	15.91	0.016	0.953
Propionic acid, g/kg DM	0.29	0.29	0.29	0.000	0.971
Butyric acid, g/kg DM	0.07^a^	0.07^a^	0.01^b^	0.004	< 0.0001*

Variables	Levels				SEM	*p*
	0	500	1000	2000		
T, °C after opening the silo	23.63	24.18	24.20	23.95	0.029	0.81
pH after opening the silo	4.41	4.64	4.56	4.45	0.012	0.003^†^
NH_3_‐N, g/kg CP	0.65	0.74	0.72	0.74	0.004	0.36
Lactic acid, g/kg DM	86.53	70.40	72.41	76.89	0.744	0.018^†^
Acetic acid, g/kg DM	17.35	16.61	14.94	15.38	0.131	0.141
Propionic acid, g/kg DM	0.32	0.30	0.28	0.28	0.002	0.179
Butyric acid, g/kg DM	0.06	0.05	0.05	0.05	0.0004	0.098

*Note:* 0 = control (0 mg); 500 = 500 mg/kg of mass forage; 1000 = 1000 mg/kg of mass forage; 2000 = 2000 mg/kg of mass forage.

Abbreviations: NH_3_‐N, ammoniacal nitrogen; SEM, standard error of the mean; T, temperature.

* Means followed by different letters in a line differ significantly by the Tukey test (*p* < 0.05).

^†^ Regression analysis. pH = 4.08 ± 0.11 + 0.42 ± 0.10 × Levels − 0.08 ± 0.02 × Levels^2^; Lactic Acid = 89.78 ± 4.20 − 5.29 ± 1.53 × Levels.

Gas and effluent losses, as well as DMR and microbial populations, were not affected by essential oil type (*p* > 0.05) (Table 3). However, increasing the inclusion levels of essential oil resulted in a linear decrease in DMR (*p* = 0.001) and a linear increase in LAB counts (*p* = 0.016) (Table 3).

**TABLE 3:** Effects of essential oils and different levels on losses, dry matter recovery, and microbial counts of sorghum silage.

Variables	Essential oils			SEM	*p*
	Tea tree	Citronella	Rosemary		
Gas losses, g/kg DM	47.26	40.50	42.30	4.148	0.806
Effluent losses, g/kg DM	7.13	8.59	8.71	1.699	0.962
Dry matter recovery, g/kg DM	945.61	950.91	948.99	9.208	0.731
Flieg's Score	75.17	79.28	79.94	0.285	0.342
LAB, log_10_/g fresh silage	7.47	7.51	7.44	0.035	0.826
Enterobacteria, log_10_/g fresh silage	2.97	3.07	2.68	0.104	0.435
Fungi, log_10_/g fresh silage	7.37	7.09	7.25	0.041	0.114

Variables	Levels				SEM	*p*
	0	500	1000	2000		
Gas losses, g/kg DM	42.62	42.47	45.58	45.74	3.612	0.993
Effluent losses, g/kg DM	4.08	4.12	4.03	4.59	0.896	0.990
Dry matter recovery, g/kg DM	953.30	953.41	950.39	948.67	1.556	0.001^†^
Flieg's Score	77.62	72.19	79.70	83.01	0.465	0.07
LAB, log_10_/g fresh silage	7.24	7.56	7.49	7.41	0.004	0.016^†^
Enterobacteria, log_10_/g fresh silage	3.18	2.57	2.89	2.98	0.182	0.398
Fungi, log_10_/g fresh silage	7.16	7.46	7.08	7.25	0.748	0.083

*Note:* 0 = control (0 mg); 500 = 500 mg/kg of mass forage; 1000 = 1000 mg/kg of mass forage; 2000 = 2000 mg/kg of mass forage.

Abbreviations: LAB, lactic acid bacteria; SEM, standard error of the mean.

^†^ Regression analysis. Dry matter recovery = 954.59 ± 6.66 − 0.004 ± 0.001 × Levels; LAB = 7.26 ± 0.10 + 0.085 ± 0.036 × Level.

**FIGURE 1:**
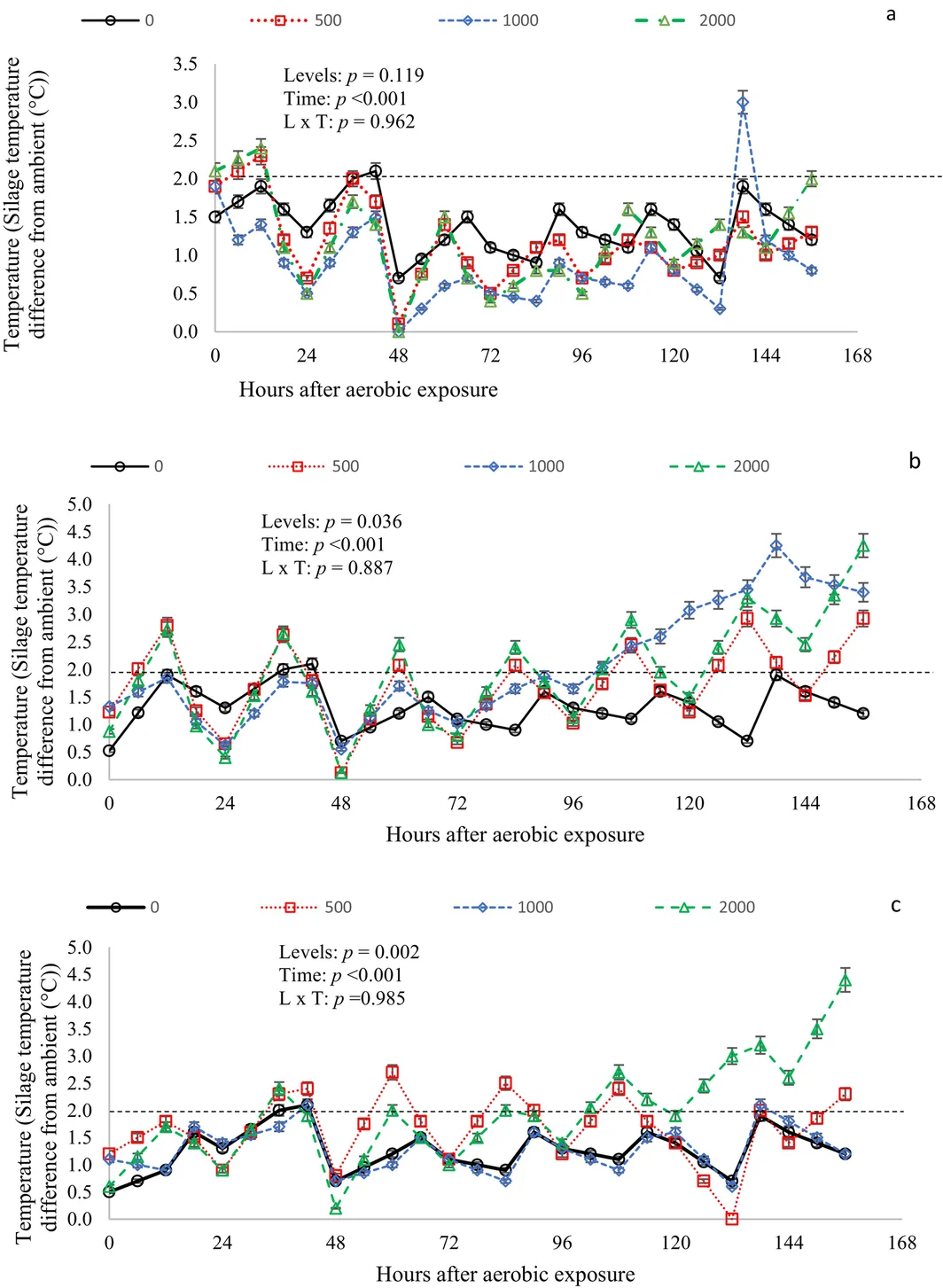
Sorghum silage temperature with different levels of essential oils for 7 days, expressed as means and standard error. 0 = Control (0 mg); 500 = 500 mg/kg of forage mass; 1000 = 1000 mg/kg of forage mass; 2000 = 2000 mg/kg of forage mass. (a) Tea tree, (b) citronella, and (c) rosemary.

**FIGURE 2:**
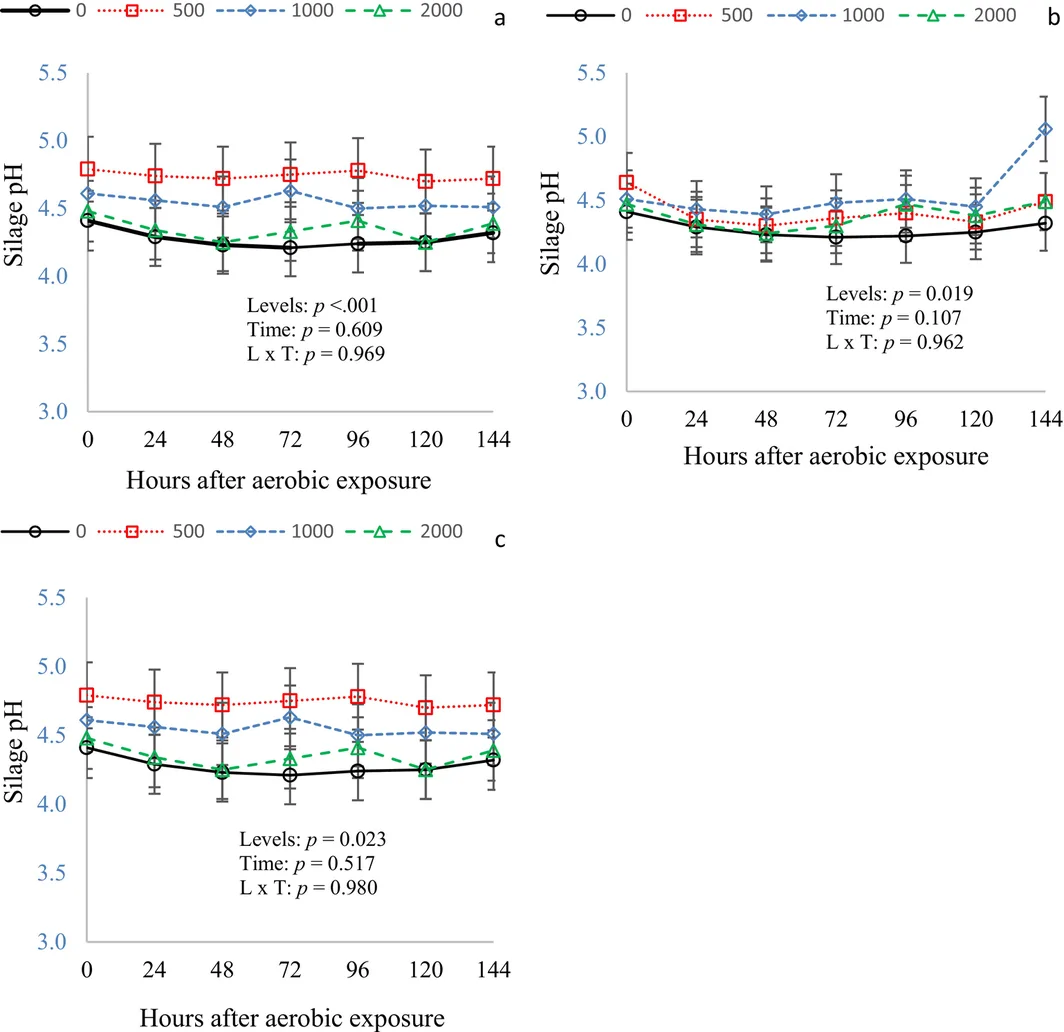
Sorghum silage pH with different levels of essential oils for 6 days, expressed as means and standard error. 0 = Control (0 mg); 500 = 500 mg/kg of forage mass; 1000 = 1000 mg/kg of forage mass; 2000 = 2000 mg/kg of forage mass. (a) Tea tree, (b) citronella, and (c) rosemary.

Regarding aerobic stability, citronella and rosemary essential oils influenced (*p* < 0.05) the duration of stability under air exposure (Figure 1b,c, respectively). Although the interaction between essential oil source and inclusion level was not significant, dose–response relationships were evaluated separately within each essential oil source during aerobic exposure. Under these conditions, silages treated with 1000 mg kg^−1^ of citronella essential oil exhibited prolonged aerobic stability (*p* = 0.036), extending the stability period by 96 h compared with the other inclusion levels (Figure 1b). Additionally, essential oil treatments affected (*p* < 0.05) pH dynamics during aerobic exposure (Figure 2). In particular, the inclusion of 500 mg kg^−1^ of tea tree and rosemary essential oils resulted in higher pH values during the evaluation period (Figure 2a,c).

## 4 Discussion

The observed increase in CP content in silages treated with rosemary essential oil (Table 1) may be associated with changes in fermentation patterns during the ensiling process. The lower NH_3_‐N concentration observed in silages treated with rosemary essential oil suggests reduced proteolysis. However, the specific microbial groups responsible for this effect were not evaluated in the present study. Therefore, the antimicrobial properties of rosemary essential oil and its antioxidant activity may have contributed to improved nitrogen preservation. In this context, the 13.15% reduction in NH_3_‐N concentration from rosemary essential oil compared with citronella essential oil (Table 2) suggests a lower extent of protein degradation. Microbial groups such as enterobacteria and clostridia are known to contribute to proteolysis and ammonia production during ensiling through amino acid deamination and protein degradation (Tuovinen et al. 2025). Similar reductions in NH_3_‐N have been reported following the application of essential oil compounds, which have been associated with decreased deamination activity during ensiling (Foskolos et al. 2016). In addition, rosemary essential oil exhibits antioxidant properties, largely attributed to compounds such as 1,8‐cineole (Nieto et al. 2018). These compounds have been reported to neutralize reactive oxygen species and interrupt free radical chain reactions. Although oxidative protein damage was not evaluated in the present study, these antioxidant properties may represent a potential explanation for improved nitrogen preservation (Lobo et al. 2010). Therefore, the antimicrobial and antioxidant activities of rosemary essential oil should be considered as possible mechanisms that may have contributed to the improved nitrogen conservation observed in silages treated with it.

A pronounced reduction in butyric acid concentration (85.7%) was observed in silages treated with rosemary essential oil compared to the other treatments (Table 2), indicating a strong inhibitory effect on clostridial activity. The production of butyric acid is typically associated with the metabolic activity of *Clostridium* spp., which utilize lactic acid as a substrate, leading to secondary fermentation and a consequent increase in silage pH (Pahlow et al. 2003). However, *Clostridium* populations were not evaluated in the present study, and therefore the specific microorganisms responsible for the observed reduction in butyric acid could not be identified. Furthermore, the relatively high dry matter content of the forage at ensiling may also have limited clostridial activity, contributing to the low butyric acid concentrations observed. Although not statistically significant, the tendency for differences in pH among treatments (*p* = 0.073) suggests that this mechanism may have influenced the fermentation process. The presence of *Clostridium* spp. is also associated with increased NH_3_‐N concentrations and reductions in CP content, as reported by Kung Jr et al. (2018), consistent with the trends observed in this study (Table 2). Nevertheless, the butyric acid concentrations remained below 1%, a threshold commonly associated with well‐preserved silages (Kung Jr et al. 2018), indicating that fermentation quality was not severely compromised. Despite pH values exceeding 4.0 in this study, silage pH responded quadratically to increasing levels of essential oil inclusion (Table 2). In contrast, lactic acid concentration decreased linearly with increasing levels of essential oil inclusion. Lactic acid is the primary acid responsible for silage acidification due to its stronger acid dissociation constant (p*K*_a_ = 3.86) compared with acetic acid (p*K*_a_ = 4.75) and propionic acid (p*K*_a_ = 4.87) (Kung Jr et al. 2018). Although not significant (*p* = 0.36), NH_3_‐N is associated with increased buffering capacity resulting from proteolysis, because ammonia is a weak base; its accumulation binds free hydrogen ions, thereby directly resisting the drop in pH and increasing buffering capacity in the silage (Fijałkowska et al. 2015). The antimicrobial activity of essential oils is known to depend on both their chemical composition and inclusion level. Previous studies have demonstrated that compounds such as carvacrol, thymol, eugenol, and cinnamaldehyde can inhibit fungi but may also negatively affect LAB (Souza 2021; Bolsen et al. 1996; Chen et al. 2023). In the present study, however, no differences were observed among essential oil sources for LAB populations, suggesting limited inhibitory effects on these microorganisms. Conversely, LAB counts increased linearly with increasing essential oil inclusion levels (Table 3), indicating that the essential oils did not exert inhibitory effects on these microorganisms under the conditions of the present study. This response should not be interpreted as a specific protective effect of the essential oils on LAB, but rather as evidence that the evaluated inclusion levels were not detrimental to LAB viability under the conditions of this study. In contrast, the lactic acid concentration decreased linearly with increasing essential oil inclusion (Table 2). This apparent dissociation between LAB abundance and lactic acid concentration suggests that LAB counts alone may not fully reflect fermentation dynamics. This dissociation suggests that LAB counts alone may not fully reflect fermentation dynamics, since microbial abundance does not necessarily correspond to metabolic activity (Yan et al. 2026). A possible explanation is that essential oils affected the metabolic activity of LAB without substantially reducing their viable populations (Silva et al. 2025). Alternatively, the essential oils may have modified the composition of the LAB community, although this hypothesis could not be evaluated because only total LAB counts were determined. Further studies using culture‐independent molecular techniques are needed to clarify the mechanisms underlying this response. However, lactic acid is essential for successful ensiling, as it suppresses undesirable microorganisms by acidifying the medium (Muck 2010; Kung Jr et al. 2018). Although some degree of dry matter loss is unavoidable during fermentation, appropriate management practices can minimize these losses and preserve silage quality (Borreani et al. 2018). In this study, the inclusion of essential oils at 2000 mg kg^−1^ resulted in a slight reduction in dry matter recovery (0.5%); however, all values remained above 94%, indicating limited impact on overall conservation efficiency.

Aerobic stability is commonly defined as the ability of silage to resist deterioration following silo opening, reflecting the rate at which spoilage processes develop upon exposure to oxygen. This parameter is closely associated with increases in temperature and shifts in pH (Kung Jr et al. 2018). Once oxygen becomes available, aerobic microorganisms, particularly yeasts, filamentous fungi, and aerobic bacteria, rapidly proliferate and metabolize organic acids, mainly lactic acid, as energy substrates. This metabolic activity results in heat production, leading to a rise in silage temperature, while the depletion of organic acids contributes to a progressive increase in pH (Borreani et al. 2018). Sorghum silage is particularly susceptible to aerobic deterioration because higher concentrations of fermentable substrates and lower levels of inhibitory compounds, commonly found in well‐preserved silages, can promote the proliferation of spoilage microorganisms (Wilkinson and Davies 2013). In the present study, the inclusion of citronella essential oil at 1000 mg kg^−1^ of fresh forage extended the aerobic stability period (Figure 1b). This response is likely related to the antifungal activity of its major constituents, particularly β‐citronellal and geraniol. Essential oils are known to disrupt key physiological processes in fungal cells, including respiratory pathways, protein synthesis, and DNA replication, while also promoting oxidative stress (Nazzaro et al. 2013). These mechanisms may limit fungal proliferation during aerobic exposure, thereby delaying silage deterioration. Regarding pH dynamics, the inclusion of citronella essential oil at 500 mg kg^−1^ maintained silage pH within 4.3–4.6 for up to 144 h of air exposure (Figure 2b), indicating a stabilizing effect during the aerobic phase. Overall, factors such as low oxygen availability prior to silo opening, controlled temperature, and acidic conditions are essential for maintaining silage stability, as they restrict the growth of undesirable microorganisms, including clostridia, yeasts, and molds (McDonald et al. 1991; Muck et al. 2018; Ávila and Carvalho 2019).

The effects of the essential oils depended on the evaluated fermentation parameter. Rosemary essential oil was the most effective in reducing proteolysis and butyric acid concentration, whereas citronella essential oil at 1000 mg kg^−1^ provided the greatest improvement in the aerobic stability of sorghum silage. The inclusion of essential oils did not significantly affect fermentative losses.

Overall, increasing essential oil inclusion levels promoted a linear increase in LAB counts. The 500 mg kg^−1^ inclusion level was associated with favorable crude protein content, while the 1000 mg kg^−1^ inclusion level provided the best overall response in terms of aerobic stability.

## Author Contributions

**José Ribeiro Meirelles Júnior:** investigation, methodology, writing – original draft. **Tadeu Silva de Oliveira:** conceptualization, data curation, investigation, formal analysis, supervision, funding acquisition, writing – review and editing, writing – original draft, resources. **Ismael Nacarati da Silva:** conceptualization, investigation. **Elvanio José Lopes Mozelli Filho:** methodology. **Elon Souza Aniceto:** writing – review and editing. **Malony Tavares Lima:** methodology. **Jéssica Luizi de Souza Andrade:** methodology. **Breno Augusto Vaz Santos:** methodology.

## Funding

This work was supported by Fundação Carlos Chagas Filho de Amparo à Pesquisa do Estado do Rio de Janeiro (E‐26/204.325/2025), Conselho Nacional de Desenvolvimento Científico e Tecnológico, Coordenação de Aperfeiçoamento de Pessoal de Nível Superior, and Universidade Estadual do Norte Fluminense (SEI‐260002/006970/2024).

## Conflicts of Interest

The authors declare no conflicts of interest.

## Data Availability

The data that support the findings of this study are available from the corresponding author upon reasonable request.
